# Factors influencing the quality of life in survivors of differentiated thyroid cancer based on patient-reported outcomes: a single-center cross-sectional study

**DOI:** 10.3389/fendo.2025.1565633

**Published:** 2025-04-30

**Authors:** Zhizhong Dong, Xiangxiang Zhan, Wen Liu, Dewei Rao, Miao Yang, Ying Peng, Yanjun Su, Ruochuan Cheng

**Affiliations:** Department of Thyroid Surgery, Clinical Research Center for Thyroid Diseases of Yunnan Province, The First Affiliated Hospital of Kunming Medical University, Kunming, China

**Keywords:** thyroid cancer, quality of life, postoperative complaints, follow up, survivorship

## Abstract

**Purpose:**

While the prognosis for differentiated thyroid cancer (DTC) is favorable, the health-related quality of life (QOL) of survivors is not well understood. This study aims to investigate the factors influencing the QOL of DTC survivors.

**Methods:**

A total of 860 DTC survivors who underwent thyroidectomy were surveyed. Participants completed the Chinese version of the Thyroid Cancer-Specific Quality of Life (THYCA-QOL) questionnaire, the European Organization for Research and Treatment of Cancer Quality of Life Core Questionnaire-C30 (EORTC QLQ-C30), and additional related questions. Multivariate regression analyses identified factors affecting survivors’ QOL.

**Results:**

Among the survivors, 65 patients (7.6%) reported long-term postoperative complaints, including fatigue, throat discomfort, neck/shoulder stiffness, weight gain, and insomnia, among others. The average THYCA-QOL summary score was 20.29, with the highest scores in problems with scar, psychological problems, gained weight, less interest in sex, and sympathetic problems. The average EORTC QLQ-C30 summary score was 82.59, with lower scores for emotional and cognitive on the functional scales, and higher scores for fatigue and insomnia on the symptom scales. Women, BMI ≥ 28, higher T-stage (T3 + 4), permanent hypoparathyroidism, recurrence reoperation, and more postoperative complaints were associated with poorer thyroid cancer-specific QOL, while age over 45 years was associated with better QOL. Longer postoperative follow-up (>6 months) and drinking were correlated with higher QLQ-C30 summary scores, while recurrence reoperation and postoperative complaints were associated with worse QOL.

**Conclusions:**

The QOL of DTC survivors is influenced by multiple factors, with some patients experiencing long-term complaints. Attention to the QOL and postoperative complaints in DTC survivors is essential.

## 1 Introduction

Over the past two decades, the incidence of thyroid cancer has surged globally. According to Global Cancer Statistics, the annual number of thyroid cancer cases increased twelvefold between 2002 and 2020, while the mortality rate remained relatively stable (1, 2). In China, the incidence of thyroid cancer has grown at a rate of 20% annually in recent years (3). This rapid increase is largely attributed to the widespread use of neck ultrasonography, which has led to the early detection of small papillary thyroid cancers (4). Differentiated thyroid cancer (DTC) is the most common type of primary thyroid malignancy, accounting for more than 90% of all thyroid cancer cases (5). The five-year survival rate for thyroid cancer is as high as 98% (6), and although the prognosis is extremely favorable, studies have shown that the overall quality of life (QOL) of thyroid cancer survivors is similar to that of survivors of other cancers, such as colon cancer, glioma, and gynecologic cancers (7). This suggests that the effectiveness of cancer treatment should not be judged solely by prognosis.

Both thyroid cancer itself and treatment-related interventions can lead to a series of physical and psychological issues for patients, such as fear, anxiety, postoperative voice changes, numbness in the hands and feet, surgical scars, and complications following radioactive iodine therapy, all of which may affect their QOL. Given that DTC patients have nearly the same life expectancy as the general population, enhancing their postoperative QOL should be given greater attention. The 2015 ATA guidelines also emphasize that physicians should consider the long-term QOL when making treatment decisions (5). Therefore, understanding the QOL of thyroid cancer patients after surgery is of utmost importance.

Given the limited data on postoperative QOL of patients with DTC, and considering that health-related QOL reports from thyroid cancer survivors can further guide surgeons in treatment and management decisions. We used the Chinese version of the Thyroid Cancer-Specific Quality of Life (THYCA-QOL) questionnaire in conjunction with the European Organization for Research and Treatment of Cancer Quality of Life Questionnaire-C30 (EORTC QLQ-C30) to assess relevant factors affecting the QOL of Chinese DTC survivors.

## 2 Materials and methods

### 2.1 Patients and study design

This study is a single center cross-sectional survey based on the thyroid tumor patient database established at our center. The database includes demographic and clinicopathological data of patients who have undergone thyroid surgery at our center. Additionally, we set up a follow-up group chat via the WeChat application early on to actively recruit postoperative patients, facilitating effective follow-up management. The survey was conducted by dedicated researchers who distributed online questionnaires within the follow-up group, explaining the study’s purpose and guiding participants on how to complete the questionnaire. Researchers assisted in clarifying any questions for patients who had difficulty understanding them but refrained from interfering with their responses. Patients who voluntarily participated in the study completed the questionnaire survey through an online electronic link and were considered participants. The survey was conducted from September 4 to November 8, 2021. Participants who met the following criteria were included in the study (1): underwent surgery with a pathological diagnosis of DTC, (2) aged >18 years. Exclusion criteria included: (1) comorbidities with other cancers, (2) history of psychiatric disorders or cognitive dysfunction, (3) incomplete questionnaires or missing data. The study was approved by the Ethics Committee of the First Affiliated Hospital of Kunming Medical University (2017 L No. 17). All participants provided informed consent.

### 2.2 Data collection

Retrospective collection of participant data from the database, including gender, age at diagnosis, marital status, employment status, smoking, drinking, family history, body mass index (BMI), surgical approach, tumor pathological characteristics, tumor staging, radioiodine (RAI) treatments, reoperation due to recurrence, and thyroid function, PTH, and blood calcium levels during the month prior to completing the questionnaire. The comorbidities of the patients mainly consisted of chronic diseases, including hypertension, diabetes, asthma, coronary heart disease, and gastrointestinal ulcers. All surveyed patients underwent conventional open surgery, with varying lengths of neck incisions resulting from differences in the severity of the primary lesion and the extent of cervical lymph node dissection. The questionnaire included the following content: 1) whether long-term calcium or/and vitamin D supplementation after surgery; 2) whether accompanied by long-term discomfort or complaints after surgery; 3) QOL Questionnaire: Chinese version of THYCA-QOL and EORTC QLQ-C30. Permanent hypoparathyroidism is defined as postoperative PTH, blood calcium levels below the normal reference range, and hypocalcemia symptoms that require calcium supplements and vitamin D treatment for more than 6 months. Evaluate the disease status of survivors with biochemical incomplete response and structural incomplete response according to the 2015 ATA guidelines (5).

The THYCA-QOL consists of 7 symptom scales (neuromuscular, voice, concentration, throat/mouth, sympathetic, psychological and sensory problems) and 6 separate items (problems with scar, chills, tingling hands/feet, weight gain, headache, and interest in sex), totaling 24 items. The sexual interest question refers to the past four weeks, while all other questions refer to the past week. Each item is rated on a four-point scale: not at all, a little, quite a bit, and very much, corresponding to scores of 1, 2, 3, and 4, respectively. The THYCA-QOL summary score is represented by the average score of all items.

The EORTC QLQ-C30 consists of 5 functional scales (physical, cognitive, role, emotional, and social), 3 symptom scales (pain, fatigue, and nausea/vomiting), 6 separate items (dyspnea, insomnia, appetite loss, constipation, diarrhea, and financial difficulties), and global health status, totaling 30 items. All questions refer to the past week. The two questions about global health status are classified on a seven-point scale, corresponding to scores ranging from 1 to 7. The remaining questions are classified on a four-point scale: not at all, a little, quite a bit, and very much, corresponding to scores of 1, 2, 3, and 4, respectively. A summary score was calculated from 13 scales (excluding global health status and financial difficulties) with the symptom scales being reversed (100 - symptom scale) to obtain a uniform direction of all scales (8–10), as follows: EORTC QLQ-C30 summary score = (physical functioning + role functioning + social functioning +  emotional functioning  + cognitive functioning  + (100-fatigue) + (100-pain) +  (100-nausea/vomiting) +  (100-dyspnea) + (100-insomnia) + (100-appetite loss) + (100-constipation) + (100-diarrhea))/13.

All question items of the THYCA-QOL and EORTC QLQ-C30 are converted into scale scores ranging from 0 to 100. Higher scores on the functional scales reflect a better QOL, whereas higher scores on the symptom scales indicate a poorer QOL.

### 2.3 Statistical analysis

The IBM SPSS version 26.0 was used for all the statistical analyses. Continuous variables are presented as mean ± standard deviation or median (interquartile range, IQR). Categorical variables are presented as numbers with corresponding percentages. Univariate analysis of the QOL summary score and patient characteristics was conducted using the Mann-Whitney U test. Variables with a *P* < 0.1 from the univariate analysis were selected for stepwise multiple linear regression analysis. The variance inflation factor (VIF) < 5 was used to assess potential multicollinearity. Statistical significance was defined as *P* < 0.05.

## 3 Results

### 3.1 Patient characteristics

A total of 922 thyroid cancer survivors participated in this survey, among which 44 cases with benign tumors and 3 cases with medullary thyroid cancer were excluded. Among the 875 patients with DTC, 13 questionnaires were incomplete, and 2 patients under the age of 18 were excluded. Ultimately, 860 DTC survivors were included in the analysis, resulting in an effective response rate of 93.3%.

The average age of the survivors was 42 years, with 707 (82.2%) being female. The median postoperative follow-up time was 15.0 months (range: 1–118 months), and the average TSH level was 1.17 μIU/ml. Papillary thyroid carcinoma (PTC) was the most common pathological type in this study, accounting for 99.1%. A total of 468 patients (54.4%) underwent total thyroidectomy, and 52 patients (6.0%) received postoperative RAI treatment. Only a few patients developed permanent hypoparathyroidism (2.5%), biochemical incomplete response (3.0%), structural incomplete response (2.2%), and recurrence reoperation (2.1%) after surgery. Sixty-five patients (7.6%) experienced one or more serious postoperative complaints for a long time after surgery, including fatigue (n=15, 1.7%), foreign body sensation in the throat (n=13, 1.5%), neck or shoulder stiffness/tightness (n=12, 1.4%), weight gain (n=9, 1.0%), insomnia (n=9, 1.0%), numbness in hands and feet (n=8, 0.9%), hair loss (n=6, 0.7%), headache (n=6, 0.7%), scar hypertrophy (n=5, 0.6%), hoarseness (n=3, 0.3%), palpitations (n=2, 0.2%), and constipation (n=1, 0.1%). **Table 1** presents the demographic and clinical pathological characteristics of the DTC survivors.

**Table 1:** Patient characteristics.

Characteristics	Total, N=860(%)
Gender	
Men	153(17.8%)
Women	707(82.2%)
Age at diagnosis (years)	42.0 ± 9.3
18-44	524(60.9%)
45-59	303(35.2%)
≥60	33(3.8%)
Follow- up duration (months)	15.0(8.0-28.0)
≤6	171(19.9%)
7-24	428(49.8%)
25-60	219(25.5%)
>60	42(4.9%)
BMI (kg/m^2^)	23.5 ± 3.3
<24	509(59.2%)
24-28	267(31.0%)
≥28	84(9.8%)
Ethnicity	
Han	726(84.4%)
Other	134(15.6%)
Marital status	
Married	773(89.9%)
Single^*^	87(10.1%)
Employment status	
Employed	652(75.8%)
Unemployed	208(24.2%)
Chronic disease	
Yes	158(18.4%)
No	702(81.6%)
Residence	
Urban	763(88.7%)
Rural	97(11.3%)
Smoking	
Yes	66(7.7%)
No	794(92.3%)
Drinking	
Yes	23(2.7%)
No	837(97.3%)
Family history of thyroid cancer	
Yes	44(5.1%)
No	816(94.9%)
Type of thyroidectomy	
Total thyroidectomy	468(54.4%)
Partial thyroidectomy^†^	392(45.6%)
Central lymph node dissection	
Bilateral	450(52.3%)
Single	406(47.2%)
No	4(0.5%)
Lateral neck lymph node dissection	
Yes	114(13.3%)
No	746(86.7%)
Parathyroid autotransplantation	
Yes	134(15.6%)
No	726(84.4%)
Thyroid cancer type	
Papillary thyroid cancer	852(99.1%)
Thyroid follicular cancer	8(0.9%)
T	
T1	723(84.1%)
T2	31(3.6%)
T3	77(9.0%)
T4	29(3.4%)
N	
N0	486(56.5%)
N1a	282(32.8%)
N1b	92(10.7%)
TNM	
I	834(97.0%)
II	22(2.6%)
III	4(0.5%)
Hashimoto thyroiditis	
Yes	228(26.5%)
No	632(73.5%)
Multifocality	
Yes	327(38.0%)
No	533(62.0%)
Recurrence risk	
Low risk	729(84.8%)
Intermediate risk	86(10.0%)
High risk	45(5.2%)
Current TSH level (μ IU/mL)	1.17 ± 1.17
≤0.5	265(30.8%)
0.5-2	467(54.3%)
>2	128(14.9%)
Biochemical incomplete response	
Yes	26(3.0%)
No	834(97.0%)
Structural incomplete response	
Yes	19(2.2%)
No	841(97.8%)
Recurrence reoperation	
Yes	18(2.1%)
No	842(97.9%)
RAI treatment	
Yes	52(6.0%)
No	808(94.0%)
Permanent hypoparathyroidism^Δ^	
Yes	17(2.5%)
No	672(97.5%)
Calcium or/and Vitamin D intake	
Yes	268(31.2%)
No	592(68.8%)
Postoperative complaints	
0	795(92.4%)
1	48(5.6%)
≥2	17(2.0%)

^*^Including divorced, widowed and never married.

^†^Including isthmectomy and lobectomy.

^Δ^The incidence of permanent hypoparathyroidism was assessed based on 689 DTC patients with a postoperative follow-up period exceeding 6 months.

BMI, body mass index; TSH, thyrotropin; RAI, radioiodine.

### 3.2 QOL score

The THYCA-QOL and EORTC QLQ-C30 scores are shown in **Table 2**. The mean THYCA summary score was 20.29, with the highest scores observed in the following five areas: Problems with scar (27.13), Psychological problems (26.85), Gained weight (25.04), Less interest in sex (23.26), and Sympathetic problems (21.36), all of which were above the average summary score. The mean QLQ-C30 summary score was 82.59, with the average Global health status score at 70.89. Among the functional scales, Emotional functioning (69.73) and Cognitive functioning (70.58) had relatively lower scores, while Fatigue (26.93) and Insomnia (26.82) had relatively higher scores on the symptom scales.

**Table 2:** THYCA-QOL and EORTC QLQ-C30 scores.

THYCA-QOL	Mean ± SD	EORTC QLQ-C30	Mean ± SD
THYCA summary score	20.29 ± 11.47	QLQ-C30 summary score	82.59 ± 13.73
Neuromuscular problems	18.13 ± 15.78	Global health status	70.89 ± 19.33
Voice problems	16.49 ± 22.04	Physical functioning	79.93 ± 28.49
Concentration problems	18.62 ± 19.54	Role functioning	84.26 ± 31.02
Sympathetic problems	21.36 ± 21.17	Emotional functioning	69.73 ± 27.92
Throat/mouth problems	16.06 ± 16.26	Cognitive functioning	70.58 ± 27.69
Psychological problems	26.85 ± 19.25	Social functioning	79.46 ± 31.10
Sensory problems	19.88 ± 18.62	Fatigue	26.93 ± 20.39
Problems with scar	27.13 ± 30.86	Nausea/vomiting	3.35 ± 9.35
Felt chilly	19.92 ± 25.50	Pain	10.66 ± 15.72
Tingling hands/feet	12.36 ± 21.36	Dyspnea	16.05 ± 20.11
Gained weight	25.04 ± 28.15	Insomnia	26.82 ± 27.75
Headache	18.64 ± 21.99	Appetite loss	6.67 ± 14.72
Less interest in sex	23.26 ± 22.15	Constipation	13.06 ± 20.63
		Diarrhea	6.78 ± 14.98
		Financial difficulties	9.61 ± 19.52

### 3.3 Univariate analysis of QOL summary score and patient characteristics

The DTC diagnosis age, gender, type of thyroidectomy, T-stage, postoperative recurrence risk stratification, recurrence reoperation, RAI treatment, permanent hypoparathyroidism, and postoperative complaints were significantly correlated with the THYCA-QOL summary score. Postoperative duration, BMI, gender, Hashimoto thyroiditis, recurrence reoperation, and postoperative complaints were significantly correlated with the QLQ-C30 summary score (P < 0.05). The relationship between QOL summary scores and patient characteristics is shown in **Table 3**.

**Table 3:** The relationship between THYCA-QOL summary score, EORTC QLQ-C30 summary score, and patient characteristics.

	THYCA-QOL summary score (Mean ± SD)	*P*	EORTC QLQ-C30 summary score (Mean ± SD)	*P*
Gender		<0.001		0.015
Men	16.96 ± 10.66		84.88 ± 12.89	
Women	21.01 ± 11.52		82.09 ± 13.87	
Age at diagnosis (years)		0.006		0.660
18-44	21.30 ± 11.79		82.26 ± 13.78	
45-59	18.93 ± 10.88		82.94 ± 13.89	
≥60	16.71 ± 9.63		84.48 ± 11.48	
Follow- up duration (months)		0.405		<0.001
≤6	20.41 ± 11.59		66.07 ± 13.79	
7-24	19.99 ± 11.42		86.29 ± 10.57	
25-60	20.29 ± 11.56		87.50 ± 9.46	
>60	22.85 ± 11.01		86.48 ± 9.82	
BMI (kg/m^2^)		0.052		0.027
<24	20.28 ± 11.22		82.19 ± 13.90	
24-28	19.50 ± 11.42		84.03 ± 13.47	
≥28	22.91 ± 12.77		80.41 ± 13.20	
Ethnicity		0.282		0.938
Han	20.06 ± 11.31		82.53 ± 13.84	
Other	21.52 ± 12.27		82.89 ± 13.20	
Marital status		0.949		0.942
Married	20.21 ± 11.34		82.77 ± 13.36	
Single^*^	20.97 ± 12.59		81.00 ± 16.65	
Employment status		0.609		0.571
Employed	20.46 ± 11.67		82.75 ± 13.54	
Unemployed	19.75 ± 10.81		82.07 ± 14.34	
Chronic disease		0.804		0.524
Yes	20.23 ± 11.75		82.11 ± 13.59	
No	20.30 ± 11.41		82.70 ± 13.77	
Residence		0.633		0.099
Urban	20.23 ± 11.50		82.83 ± 13.73	
Rural	20.78 ± 11.26		80.70 ± 13.66	
Smoking		0.131		0.520
Yes	18.59 ± 11.39		83.46 ± 13.54	
No	20.43 ± 11.47		82.52 ± 13.75	
Drinking		0.281		0.083
Yes	18.28 ± 12.26		87.23 ± 11.56	
No	20.35 ± 11.45		82.46 ± 13.77	
Family history of thyroid cancer		0.682		0.628
Yes	20.95 ± 11.08		81.35 ± 14.40	
No	20.26 ± 11.49		82.65 ± 13.70	
Type of thyroidectomy		0.010		0.225
Total thyroidectomy	21.25 ± 11.96		83.34 ± 13.15	
Partial thyroidectomy^†^	19.14 ± 10.75		81.69 ± 14.36	
Central lymph node dissection		0.263		0.074
Bilateral	20.91 ± 11.97		83.57 ± 13.09	
Single	19.65 ± 10.88		81.62 ± 14.27	
No	16.45 ± 9.07		70.48 ± 19.66	
Lateral neck lymph node dissection		0.742		0.676
Yes	20.72 ± 11.85		83.41 ± 12.99	
No	20.23 ± 11.41		82.46 ± 13.85	
Parathyroid autotransplantation		0.657		0.995
Yes	20.64 ± 11.94		82.24 ± 14.65	
No	20.23 ± 11.39		82.65 ± 13.56	
T		0.003		0.754
T1	19.77 ± 11.10		82.35 ± 14.00	
T2	18.85 ± 11.23		85.39 ± 11.46	
T3	25.17 ± 13.19		83.14 ± 12.31	
T4	21.98 ± 13.17		84.16 ± 12.72	
N		0.269		0.803
N0	19.85 ± 11.55		82.10 ± 14.30	
N1a	20.82 ± 11.07		83.20 ± 12.86	
N1b	21.03 ± 12.21		83.31 ± 13.29	
TNM		0.445		0.777
I	20.36 ± 11.49		82.53 ± 13.76	
II	19.09 ± 10.98		83.88 ± 13.70	
III	13.62 ± 10.53		88.57 ± 5.89	
Hashimoto thyroiditis		0.912		0.044
Yes	20.11 ± 11.06		80.74 ± 14.70	
No	20.36 ± 11.62		83.26 ± 13.31	
Multifocality		0.698		0.254
Yes	20.18 ± 11.54		83.48 ± 13.02	
No	20.36 ± 11.43		82.04 ± 14.14	
Recurrence risk		0.029		0.685
Low risk	19.91 ± 11.31		82.53 ± 13.91	
Intermediate risk	21.46 ± 11.93		81.97 ± 13.32	
High risk	24.26 ± 12.40		84.75 ± 11.40	
Current TSH level (μ IU/mL)		0.914		0.938
≤0.5	20.05 ± 11.16		82.60 ± 13.40	
0.5-2	20.20 ± 11.13		82.56 ± 13.81	
>2	21.14 ± 13.24		82.65 ± 14.22	
Biochemical incomplete response		0.286		0.356
Yes	23.17 ± 13.89		86.23 ± 9.07	
No	20.20 ± 11.38		82.47 ± 13.84	
Structural incomplete response		0.946		0.445
Yes	21.56 ± 15.74		84.67 ± 13.09	
No	20.26 ± 11.36		82.54 ± 13.75	
Recurrence reoperation		0.045		0.018
Yes	29.64 ± 20.34		74.95 ± 15.61	
No	20.09 ± 11.14		82.75 ± 13.65	
RAI treatments		0.031		0.829
Yes	23.62 ± 12.07		82.91 ± 12.20	
No	20.08 ± 11.40		82.57 ± 13.83	
Permanent hypoparathyroidism^Δ^		0.048		0.292
Yes	29.98 ± 17.95		84.08 ± 10.70	
No	20.05 ± 11.18		86.75 ± 10.18	
Calcium or/and Vitamin D intake		0.076		0.965
Yes	21.32 ± 11.84		82.81 ± 13.31	
No	19.82 ± 11.27		82.49 ± 13.93	
Postoperative complaints		<0.001		<0.001
0	19.70 ± 11.07		83.46 ± 13.38	
1	24.52 ± 10.13		74.49 ± 13.36	
≥2	35.90 ± 18.51		64.62 ± 11.49	

^*^Including divorced, widowed and never married.

^†^Including isthmectomy and lobectomy.

^Δ^The incidence of permanent hypoparathyroidism was assessed based on 689 DTC patients with a postoperative follow-up period exceeding 6 months.

BMI, body mass index; TSH, thyrotropin; RAI, radioiodine.

### 3.4 Multiple linear regression analysis of the impact on patients’ QOL scores

We included factors with P < 0.1 from the univariate analysis of QOL summary scores and patient characteristics into a stepwise multiple linear regression model. The results indicated that female gender, obesity (BMI ≥ 28), higher T-stage (T3 + 4), permanent hypoparathyroidism, recurrence reoperation, and increased postoperative complaints were associated with poorer thyroid cancer-specific QOL scores, while older age (> 45 years) was associated with higher thyroid cancer-specific QOL scores (**Table 4**). A longer postoperative duration (> 6 months) and drinking were positively correlated with higher QLQ-C30 summary score, while recurrence reoperation and postoperative complaints led to a lower QLQ-C30 summary score (**Table 5**). All VIFs are less than 5, and no collinearity issues were observed in the model (P < 0.05 for all).

**Table 4:** Multiple linear regression analysis of factors influencing THYCA-QOL summary score.

	B	SE	t	*P*	95%CI	VIF
Constant	16.137	0.972	16.605	<0.001	14.229 to 18.044	
Women (men as reference)	4.143	0.971	4.267	<0.001	2.237 to 6.048	1.019
Age at diagnosis (18–44 as reference)						
45-59	-2.358	0.782	-3.015	0.003	-3.894 to -0.823	1.032
≥60	-4.134	1.940	-2.131	0.033	-7.942 to -0.327	1.027
BMI (<24 as reference)						
24-28	-0.033	0.836	-0.040	0.968	-1.674 to 1.608	1.103
≥28	3.153	1.250	2.522	0.012	0.699 to 5.608	1.019
T(3 + 4)(T1 + 2 as reference)	4.217	1.121	3.761	<0.001	2.017 to 6.418	1.004
Permanent hypoparathyroidism (none as reference) ^Δ^	8.467	2.745	3.085	0.002	3.078 to 13.856	1.023
Recurrence reoperation (none as reference)	7.636	2.587	2.951	0.003	2.558 to 12.713	1.014
Postoperative complaints (none as reference)						
1	4.389	1.612	2.723	0.007	1.225 to 7.553	1.013
≥2	16.080	2.651	6.067	<0.001	10.877 to 21.282	1.007

^Δ^The incidence of permanent hypoparathyroidism was assessed based on 689 DTC patients with a postoperative follow-up period exceeding 6 months.

**Table 5:** Multiple linear regression analysis of factors influencing EORTC QLQ-C30 summary score.

	B	SE	t	*P*	95%CI	VIF
Constant	67.144	0.834	80.525	<0.001	65.507 to 68.781	
Follow- up duration (≤6 as reference)						
7-24	19.751	0.966	20.443	<0.001	17.855 to 21.648	1.776
25-60	20.850	1.092	19.087	<0.001	18.706 to 22.994	1.724
>60	20.324	1.832	11.092	<0.001	16.728 to 23.921	1.187
Drinking (none as reference)	4.592	2.255	2.037	0.042	0.167 to 9.018	1.007
Recurrence reoperation (none as reference)	-8.908	2.539	-3.509	<0.001	-13.891 to -3.925	1.005
Postoperative complaints (none as reference)						
1	-5.747	1.588	-3.618	0.001	-8.864 to -2.629	1.012
≥2	-15.345	2.614	-5.871	<0.001	-20.475 to -10.215	1.007

## 4 Discussion

Although DTC has a favorable prognosis and is often referred to as a “good cancer,” there is limited understanding of the QOL in DTC survivors. In this cross-sectional study on the QOL of DTC survivors after surgery, we combined the THYCA-QOL and EORTC QLQ-C30, which are the most widely used and common questionnaires for assessing health-related QOL in patients with DTC (11). The Chinese versions of both the THYCA-QOL and EORTC QLQ-C30, when used together, have demonstrated good reliability and validity for assessing the QOL in Chinese thyroid cancer patients (12). This study found that DTC patients experience varying degrees of impaired postoperative QOL, which is related to multiple factors. A small number of patients reported long-term discomfort or complaints postoperatively.

In this study, the mean THYCA-QOL summary score was 20.29, which is lower than that reported in a recent Chinese study on DTC survivors’ QOL (mean: 34.50) (13). This discrepancy may be related to several factors, including lower rates of RAI treatment (6.0%) and lateral neck lymph node dissection (13.3%) in our study, as well as population heterogeneity. Problems with scar is the highest-scoring domain in THYCA-QOL, as it not only affects the aesthetic appearance of the patient’s neck but also leads to scar-related symptoms in 66% of postoperative patients, such as pruritus, tightening, pain, and burning sensation (14). Liu et al. (15) conducted a propensity score-matched study of 78 pairs of patients who underwent either transoral endoscopic thyroidectomy vestibular approach or conventional open thyroidectomy, finding that the transoral endoscopic thyroidectomy vestibular approach not only provided aesthetic benefits but also improved patients’ QOL. All 860 patients in our study underwent conventional open thyroidectomy, which likely contributed to the poorer ratings for problems with scar. However, with the widespread use of endoscopic thyroid surgery, patients will have more opportunities to reduce or avoid this adverse outcome. Psychological issues in cancer patients are complex and multidimensional, often stemming from the cancer itself, treatment-related complications, and lifelong disease monitoring, all of which contribute to survivors’ psychological problems and additional complaints (16). It is worth noting that we found “Gained weight” to be another area with high patient complaints. Some studies suggest that insufficient L-T4 replacement therapy after thyroid surgery could be a cause of postoperative weight gain (17, 18). However, Polotsky et al. (19) found that DTC patients who underwent TSH suppression therapy also experienced weight gain. Sohn et al. (20) reviewed 700 DTC patients and found that DTC patients experienced significant weight gain after total thyroidectomy, with an increase from 61.3 ± 10.1 kg at baseline to 61.8 ± 10.2 kg at 3 to 4 year of follow-up (P<0.01), and younger female patients showed a more pronounced increase. Postoperative weight gain in thyroid cancer patients may be the result of multiple factors, including TSH levels, diet, lifestyle, and psychological factors. More prospective studies are needed to explore the potential causes.

In the EORTC QLQ-C30 scale, the mean summary score was 82.59, which is significantly higher than the 65.93 reported by Li et al. (9), who primarily focused on the QOL of thyroid cancer patients within three months post-surgery. Emotional functioning and Cognitive functioning had the lowest scores among the functional scales, which aligns with the findings of Wang et al. (21). Studies have shown that emotional dysfunction in DTC patients is often related to concerns, anxiety, and the fear of disease recurrence (22). Furthermore, even an average of 10 years after thyroid cancer treatment, thyroid cancer survivors continue to experience distress, anxiety, and depression which do not diminish over time following surgery (23). The highest scores on the symptom scale were observed for Fatigue and Insomnia, a finding that has been reported in several studies across different cultural backgrounds (24–26). In a study that matched thyroid cancer survivors with a normal population by gender and age, it was found that thyroid cancer patients experienced higher levels of fatigue. The researchers suggested that this might be closely related to TSH imbalances and cancer-related fatigue (27).

Through multiple linear regression, we identified several risk factors for poorer QOL in thyroid cancer survivors, including female gender, obesity, higher T-stage, permanent hypoparathyroidism, reoperation due to recurrence, and more postoperative complaints. In contrast, older age, longer postoperative duration, and drinking were identified as factors that improve QOL. Female patients are the predominant group affected by thyroid cancer, and studies have shown that female survivors experience worse QOL in relation to postoperative scars, headaches, anxiety, and are more likely to develop fatigue and sleep disturbances post-surgery (28, 29). Generally, the probability of surgical complications increases with the extent of the surgery, and consequently, QOL may worsen. Although the type of surgery was related to the THYCA summary score in our study, no profound impact on QOL was observed after multivariate adjustment. Higher T-stage and reoperation due to recurrence are significant factors contributing to worse QOL. This may be attributed to the T-stage, which reflects tumor pathological characteristics, determines the surgical extent, and involves various factors related to potential postoperative outcomes. Furthermore, a prospective longitudinal study demonstrated that QOL in low-to-intermediate recurrence-risk DTC patients is not associated with surgical extent, as the QOL differences between lobectomy and total thyroidectomy patients disappeared at both 6 months and 12 months postoperatively (30). However, the relationship between surgical extent and postoperative QOL in DTC patients requires further investigation for confirmation. Permanent hypoparathyroidism is a severe complication following thyroid surgery, though its incidence is low. However, numerous studies have shown that it significantly impacts the patient’s QOL (31–34). Wu et al. (35) found that obesity was closely associated with postoperative recurrence of thyroid cancer, and other studies have shown that obesity is related to increased risk of postoperative complications in thyroid cancer (36). Wang et al. (21) also found that being obese or overweight negatively affected the postoperative QOL in 965 thyroid cancer survivors. Additionally, it is important to highlight that in our study, some DTC survivors voluntarily reported postoperative complaints, including fatigue, neck or shoulder stiffness/tightness, weight gain, sensation of a foreign body in the throat, insomnia, hair loss, scar hypertrophy, palpitations, headaches, and constipation. Moreover, this study is the first to report that these complaints persist long-term in survivors, occurring either separately or simultaneously, and are strongly linked to lower scores on both QOL scales. We have yet to determine the exact factors contributing to these complaints, which will be the focus of our future research.

A prospective longitudinal study on the QOL of DTC survivors showed that the QOL score significantly decreased at 3 months post-surgery compared to pre-surgery levels, but gradually improved thereafter, surpassing pre-surgery levels at 5 years post-surgery (37). Our findings also indicate that the QOL of patients improves with time after surgery. In age stratification (youth, middle-aged, and elderly groups), we found an inverse relationship with the THYCA-QOL summary score, meaning younger patients reported poorer thyroid cancer-specific QOL. This may be due to younger individuals shouldering more social responsibilities and being more sensitive to clinical symptoms. Interestingly, we observed that alcohol intake was associated with higher QLQ-C30 summary scores in DTC survivors. This is not an uncommon phenomenon in cancer survivors; Lucas et al. (38) reported that non-drinking breast cancer survivors had lower physical activity levels and worse physical functioning and vitality scores. Allison (39) found that alcohol consumption was linked to improvements in physical and role functioning, as well as multiple symptom issues and global QOL in head and neck cancer patients as measured by the EORTC QLQ-C30. A prospective cohort study on colorectal cancer revealed that compared to abstainers, moderate (≤7 drinks/week) and heavy alcohol consumption (>7 drinks/week) were associated with reduced anxiety and depression, as well as better health-related QOL (40). Alcohol consumption, as a lifestyle habit, likely improves patients’ psychological and emotional states. However, our study does not encourage alcohol consumption, as it remains a major risk factor for global disease burden and health deterioration (41).

Several limitations must be acknowledged. Firstly, this study is a single-center cross-sectional study with a relatively short follow-up period, which limits our ability to predict precise timepoints affecting postoperative QOL and to analyze the dynamic changes in DTC survivors’ QOL over time. Secondly, the study population predominantly consisted of low-risk PTC patients, resulting in limited applicability that may not represent a broader spectrum of DTC pathology types. Thirdly, the investigation into demographic factors and lifestyle habits influencing DTC survivors’ QOL was not comprehensive, thus hindering a complete understanding of the contributing factors. Future research will aim to address these limitations.

In conclusion, this study demonstrates that the postoperative QOL of DTC survivors is influenced by multiple factors and may be accompanied by long-term complaints. Therefore, it is crucial to emphasize standardized treatment during initial care to minimize complications. Considering that the survival period of DTC survivors is primarily unaffected, gaining a deeper understanding of their psychological concerns, health-related information, and adverse complaints is essential for healthcare providers to offer targeted support and assistance. To achieve this, healthcare teams should focus on mitigating patients’ unnecessary psychological stress through strengthened multidisciplinary collaboration, specialized follow-up platforms for timely communication, and structured postoperative health education programs.

## Data Availability

The original contributions presented in the study are included in the article/supplementary material. Further inquiries can be directed to the corresponding authors.
